# The lipid digestion behavior of oil-in-water emulsions stabilized by different particle-sized insoluble dietary fiber from citrus peel

**DOI:** 10.1016/j.fochx.2023.100831

**Published:** 2023-08-09

**Authors:** Ben Yu, Qianqian Chen, Joe M. Regenstein, Changwen Ye, Lufeng Wang

**Affiliations:** a College of Food Science and Technology, Huazhong Agricultural University, No.1 Shizishan Street, Wuhan, Hubei 430070, China; b Hubei Key Laboratory of Fruit & Vegetable Processing & Quality Control, Huazhong Agricultural University, Wuhan, Hubei 430070, China; c Shenzhen Institute of Nutrition and Health, Huazhong Agricultural University, Shenzhen, Guangdong 518000, China; d Jinxiang Economic Development Zone Food Industrial Park, Shandong 272209, China; e Department of Food Science, Cornell University, Ithaca, NY 14853, USA; f Zhengzhou Tobacco Research Institute of China National Tobacco Corporation, Zhengzhou 450001, China

**Keywords:** Citrus peel, Insoluble dietary fiber, Particle size, Lipid digestion, Emulsion

## Abstract

In this study, oil-in-water emulsions stabilized by insoluble dietary fibre from citrus peel (CIDF) exhibited an obviously delayed lipid digestion property through gastrointestinal tract (GIT) model. Our results suggested that the rate and extent of lipid digestion greatly relied on particle sizes and concentrations of CIDF, and the inhibition effect of lipolysis was markedly enhanced with decreasing particle sizes and increasing CIDF levels. Furthermore, compared with Tween80-stabilized emulsion, the maximum inhibition extent of lipolysis was 38.77% for CIDF400-stabilized one at 0.4 wt% concentration. Effects of CIDFs on lipid digestion was mainly due to the formation of protective layers around oil droplets, further blocking the entry of lipase to the internal lipids, and/or attributed to the increasing viscosity of emulsions caused by CIDFs, finally limiting the transportation of some substances in the simulated small intestine digestion. Our research would provide useful references for the application of CIDF-stabilized emulsions in low-calorie food.

## Highlights

• Different particle-sized CIDF-stabilized emulsions were fabricated under various CIDF levels.
• A simulated three-stage gastrointestinal tract model was used to study the lipid digestion of CIDF-stabilized emulsions.
• Lipid digestion rate and extent depended on particle sizes and concentrations of CIDF.
• The initial lipid digestion rate and extent were retarded in the CIDF-stabilized emulsions.

## 1 Introduction

Over the past twenty years, the population of obesity and overweight have sharply increased throughout the world due to excessive intake and ingestion of high-calorie foods (Bai et al., 2019, Feng et al., 2017). Obesity or overweight is closely associated with increased incidences of many diseases, for instance, cardiovascular disease, diabetes, hypertension, and many others (Feng et al., 2020, Kallus and Brandt, 2012, Qasim et al., 2018). Given that, many strategies to solve this problem have been invented in the past decade, mainly including preparation and application of fat replacers or substitutes in various low-fat foods (Babu et al., 2018, Li et al., 2019, Yi et al., 2014, Yu et al., 2021) and fabrication of solid particles-stabilized Pickering emulsions to retard lipid digestion behavior and delay the lipolysis process during the *in vitro* gastrointestinal tract (GIT) model, therefore reducing energy intake in the small intestine (Costa et al., 2020, Xiao et al., 2015). However, the reduction of lipid content from food would inevitably cause the disruption of its texture and organoleptic properties to some extent, finally lowering consumers' desire to such food products (Aime, Arntfield, Malcolmson, & Ryland, 2001). Therefore, an increasing research interest in the utilization of natural ingredients (such as polysaccharides and proteins) as greener and safer emulsifiers than conventional emulsifiers to manufacture Pickering emulsions has received extensive attentions from researchers in this field (Costa et al., 2020).

It is well known that proteins are natural amphiphilic molecules, for instance whey protein isolate (WPI) and soy protein isolate (SPI) that are both most commonly protein-based emulsifiers used to stabilize emulsion systems in the presence of electrostatic and steric repulsive effects (Bellesi et al., 2016, Hu et al., 2022, McClements et al., 2017). Regrettably, protein-based emulsions are usually unstable to some conditions (e.g. pH, ionic concentration and temperature), and they are extremely broken down by various protease, which finally limits their applications in food industry (He, Li, Li, Li, & Liu, 2020). However, those problems can be avoided in the presence of food-grade polysaccharide particles that will provide a high physical and energy barrier when particles are adsorbed onto the O/W interface, thus preventing emulsion droplets from coalescence (Berton-Carabin & Schroën, 2015).

Dietary fibres, as a kind of indigestible polysaccharides, are widely distributed in various plants and exhibit many excellent properties, such as thickening effect, emulsion stability, reduction of fat content, and other health-promoting benefits (de Moraes Crizel et al., 2013, He et al., 2020, Qin et al., 2016). It has been reported that dietary fibres might influence the lipid digestion behavior within GIT model, mainly through adsorbing and binding to some critical species in the small intestine, increasing the viscosity of digesta, and inactivating digestive enzymes (Chen et al., 2020, Espinal-Ruiz et al., 2014). However, dietary fibres with different molecular properties (such as molecular weight, electrical properties and particle size) and various sources tend to differ in the physicochemical and functional properties, including adsorption property, surface active, thickening effect, and emulsifying property (Qin et al., 2016). Thus, different dietary fibres would influence lipid digestion behavior via various approaches.

A previous study indicated that water-insoluble dietary fibre from bamboo shoot (BSDF) was an excellent food-grade particle stabilizer for the fabrication of O/W Pickering emulsions (He et al., 2020). It was possible that cellulose particles, kind of insoluble dietary fibre, can improve the emulsion stability via enhanced interfacial adsorption and interactions between particles, thus preventing droplets from collision and coalescence (Costa et al., 2020). Therefore, insoluble dietary fibres (IDF) were promising alternatives to substitute protein particles as food-grade stabilizers to improve the emulsion stability. However, there have been few researches focusing on the lipid digestion behavior of emulsions influenced by the particle sizes of IDF from citrus peel.

Citrus peels, one of byproducts from citrus, are rich in dietary fibre, and especially for insoluble dietary fibre. It has been reported that citrus fiber containing 74.3 wt% insoluble fibers (IDF) and 16.5 wt% soluble fibers could be used for the formation of stable O/W emulsion through its combination of Pickering effect and fiber-based network, which might be mainly attributed to the contribution from insoluble fibers part (Qi, Song, Zeng, & Liao, 2020). In addition, citrus dietary fiber was successfully used for fabrication of high-internal-phase emulsion gels through synergistically combination of other natural substances (Chen, Zhang, Li, & Sun, 2023). A previous study indicated that physicochemical properties of insoluble dietary fiber from pomelo (Citrus grandis) peel were effectively improved or modified by ball milling, and the ball-milled IDFs were successfully applicated for the preparation of oil-in-water Pickering emulsions (Xiao, Yang, Zhao, Wang, & Ge, 2022). It was also reported in our previous study that the particle size significantly influenced the physicochemical and structural properties of insoluble dietary fibre from citrus peel (CIDF) (Feng, Yu, Regenstein, & Wang, 2022). Moreover, citrus fiber containing high levels of soluble dietary fiber was able to stabilize the emulsions by high-intensity ultrasound and affected the *in vitro* lipid digestion of emulsions (Kalla-Bertholdt, Nguyen, Baier, & Rauh, 2021). Thus, to further investigate the impact of particle size of CIDF on the lipid digestion behavior of emulsions, we prepared emulsions stabilized by various particle-sized CIDF treated by superfine grinding. Then, the mean particle diameter, particle charge, rheological property and microstructure of CIDF-stabilized emulsions were characterized before and after passage through a simulated GIT model. In addition, emulsions stabilized by different concentrations of CIDF solutions were prepared to further explore the impact of emulsifier concentration on lipid digestion behavior of CIDF-stabilized emulsions. This study would provide an understanding of the role of insoluble dietary fibre in regulating lipid digestion.

## 2 Materials and methods

### 2.1 Materials

Citrus peels were purchased from Guangdong Lemont Biotechnology Co., Ltd (Guangdong, China), and citrus peel insoluble dietary fibre (CIDF) with three different particle size was prepared according to a previous method in our laboratory (Yu et al., 2022). Those three CIDFs were named as CIDF120 (74.96 μm), CIDF200 (38.31 μm) and CIDF400 (17.52 μm), respectively, and the apparent viscosity of each fraction was shown in Fig. S1 (Supplementary data). Besides, the differences of chemical components and detailed particle size distribution of each fraction have been reported in our previous study (Feng et al., 2022). Corn oil was obtained from a local supermarket (Wuhan, China). Nile red, mucin (from the porcine stomach, Type II, M2378), pepsin (from porcine gastric mucosa, P6887, ≥ 250 units/mg), porcine pancreatic lipase (Type II, L3126, 100–500 units/mg), and bile extract (porcine, B8631) were purchased from Sigma-Aldrich Chemical Co. (St. Louis, MO, USA). All the other chemicals were of analytical grade, and they were obtained from Sinopharm Chemical Reagent Co., Ltd. (Shanghai, China).

### 2.2 Experiment method

In this experiment, there were three treatment groups and one control group (Tween 80), and three concentration levels of 0.1, 0.2 and 0.4 wt% were set up for all groups, respectively.

#### 2.2.1 Preparation of initial solutions of different concentrations

Tween 80 and different particle-sized CIDF stock solutions (0.1, 0.2 and 0.4 wt%) were prepared by dispersing 0.1, 0.2 and 0.4 g of Tween 80 and powdered CIDFs into 100 g of deionized water, respectively. Subsequent, all the stock solutions were sheared for 2 min with continuous stirring (8000 rpm/min) using a high-speed homogenizer (XHF-D, Ningbo Xinzhi Biotechnology Co., Ltd, China) to obtain uniformly dispersed stock solutions.

#### 2.2.2 Preparation of CIDF-stabilized emulsions

Stock emulsions were fabricated by blending corn oil (10%, w/w) with emulsifier solution prepared in Section 2.2.1 (90%, w/w) for 2 min using a high-speed shear homogenizer. The coarse emulsions were then homogenized under the condition of 600 bar for three cycles using a high-pressure homogenizer (AH-2010, ATS Engineering Limited, China) to obtain finer emulsions. The final emulsifier level in the resulted emulsion were 0.09 wt%, 0.18 wt% or 0.36 wt%, respectively. Emulsions obtained above were placed at 4 °C fridge for the subsequent experiment.

### 2.3 A simulated gastrointestinal tract (GIT) model

In this study, a simulated GIT model, including mouth, stomach, and small intestine phases was carried referring to a previously reported study with some modifications (Bai et al., 2019). All the emulsions were subjected to a simulated GIT digestion model, and among them, Tween 80-stabilized emulsions with different concentrations were used as a reference.

#### 2.3.1 Mouth stage

The emulsion samples (20 mL) were mixed with equivalent volume of pre-heated simulated saliva fluid (SSF, 37 ℃) comprised of 0.03 g/mL porcine gastric mucin. Subsequently, NaOH solutions (0.1 M) were used to adjust the pH of the mixed system to 6.8, followed by a continuous shaking at 100 r/min in 37 °C water bath for 10 min to simulate oral phase.

#### 2.3.2 Stomach stage

Before performing *in vitro* simulated gastric digestion experiment, sodium chloride (2.0 g) and hydrochloric acid (7.0 mL) were added into deionized water, and then diluted to 1000 mL with pH adjusted to 1.2 using HCl solutions (1 M) to prepare simulated gastric fluid (SGF). Then, the emulsion samples obtained from the oral phase (20 mL) were blended with equal volume of pre-heated SGF (37 °C) containing 3.2 mg/mL pepsin, followed by the pH change from 1.2 to 2.5 using 1 M NaOH solutions. Subsequent, the mixed system was placed in 37 °C water bath with constant oscillation (2 h) to simulate the gastric digestion stage.

#### 2.3.3 Small intestine stage

The samples obtained from the gastric digestion phase (20 mL) was placed into a 100 mL glass beaker and the pH was adjusted to 7.0 using NaOH solutions, followed by the addition of 1.5 mL simulated small intestinal fluid (SIF) that contained 0.25 M CaCl_2_ and 3.75 M NaCl, and 3.5 mL of bile salt solutions (5 mg/mL). Then, the pH value of the mixture was set back to 7.0 and the reaction vessel was maintained in 37 °C water bath with continuous shaking. Subsequently, 2.5 mL lipase solution (1.6 mg/mL, 400-2000U) was added to initialize the lipolysis reaction. In the end, an automatic titration device (905 Titrando, Metrohm USA Inc., Hillsborough, FL, USA) was used to maintain the system constant pH of 7.0. In this period, NaOH solutions (0.1 M) were added dropwise into the glass beaker to neutralize free fatty acids (FFAs) released by the lipid hydrolysis reaction for 2 h automatically. The lipid digestion profiles in this period could be obtained according to the release content of FFAs over time. The release percentage of FFAs was obtained according to equation (1):

(1) $$FFA\;\left(\%\;w/w\right)=100\;\times \;\;\left(\frac{V_{\mathrm{NaOH}}\left(L\right)\times C_{\mathrm{NaOH}}\left(M\right)\times {\mathrm{MW}}_{\mathrm{Lipid}}\left(g/mol\right)}{2\times W_{\mathrm{Lipid}}\left(g\right)}\right)$$

Where, C_NaOH_ represented the concentration of NaOH solution (0.1 M), and V_NaOH_ represented the titration volume of NaOH (L) to neutralize the FFAs released (assuming that a triacylglycerols (TAG) unit was decomposed two FFA molecules), MW_Lipid_ represented the average molecular weight of corn oil (872 g/mol), W_Lipid_ represented initial mass of corn oil in the simulated small intestinal fluid (SIF).

### 2.4 Emulsion characterization

#### 2.4.1 Mean particle diameter and particle size distribution measurements

The mean particle diameter and particle size distribution (PSD) of emulsions before and after passage through the *in vitro* simulation GIT digestion were determined by a Mastersize2000 analyzer (Malvern instrument, Malvern, UK). Before performing this experiment, the emulsion samples were diluted to 0.005 wt% using phosphate buffer solution (5 mM, pH 7.0) to avoid multiple scattering effects. The refractive indices (RI) of the aqueous and oil phases were 1.330 and 1.472, respectively. Finally, the volume-weighted average diameter (*d_43_ = Σn_i_d_i_^4^/Σn_i_d_i_^3^*) was used to represent average particle diameter of the emulsion droplets.

#### 2.4.2 Particle charge (zeta-potential) measurements

The particle charges of all the emulsions before and after being exposed to the *in vitro* simulation GIT digestion were determined using a particle electrophoresis instrument (Zetasizer Nano ZS, Malvern Instruments, Worcestershire, UK). Prior to analysis, the emulsion samples were diluted to 0.005% (w/w) with phosphate buffer solution (5 mM, pH 7.0) to avoid multiple scattering effect.

#### 2.4.3 Rheological behavior measurements

The rheological behavior was carried out using a dynamic shear rheometer (TA Instruments, New Castle, DE, USA) to characterize the apparent viscosity of the emulsion samples before and after passage through the simulation GIT model. Prior to analysis, the emulsion samples were placed on the rheometer plate with a 40 mm upper parallel plate. The gap between two plates was adjusted to 500 μm. Then, the samples were equilibrated for 3 mins, and the apparent viscosity of the samples were measured at room temperature (25 °C) within a shear rate ranging from 0.01 to 200 s^−1^. Finally, the apparent viscosity (η) values were obtained from a selected shear rate of 25 s^−1^.

#### 2.4.4 Microstructure observation

The microstructures of the emulsions before and after passage through the simulation GIT model were observed by a confocal laser scanning microscope (FV3000, Olympus, Japan). Prior to observation, 1 mL of the emulsion samples were stained with 10 μL of Nile Red solutions (1 mg/mL, absolute ethanol), and the mixture was equilibrated for 10 min in the dark (25 °C). Then, the sample (∼6 μL) was dropped on a microscope slide with a glass coverslip covered. A 60 × oil immersion objective was selected to observe the microstructure of oil droplets in the emulsion samples with the excitation spectrum of 543 nm for Nile red detection. Finally, the microscopic images were obtained from the software connected to this instrument with a pixel of 1024 × 1024.

### 2.5 Statistical analysis

All the experiments were performed in triplicate for each sample. Statistical software SPSS 24.0 was used to analyze the statistical significance of the experimental results (P < 0.05, Duncan) and obtain the mean and standard deviations. All the graphs were drawn by GraphPad Prism 8.0.

## 3 Results and discussion

### 3.1 Influence of particle sizes and concentrations of CIDF on physicochemical properties of emulsions in the simulated gastrointestinal tract (GIT) model

**Fig. 1:**
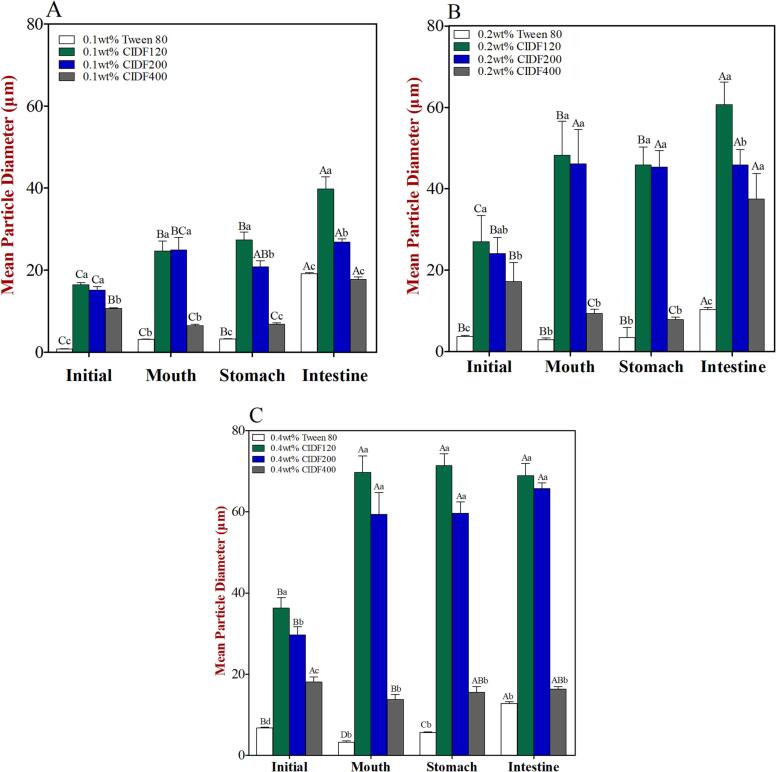
Volume-weighted mean particle diameter (d_43_) of emulsions containing different emulsifiers and various concentration levels [(a) 0.1 wt%, (b) 0.2 wt%, (c) 0.4 wt%] after being exposed to different phases of a simulated GIT model. Different capital letters (*A* to *D*) between different GIT stages (same emulsifier) were significantly different (Duncan, *p* < 0.05), and different lower case letters (*a* to *d*) between different emulsifiers (same GIT stage) were significantly different (Duncan, *p* < 0.05).

**Fig. 2:**
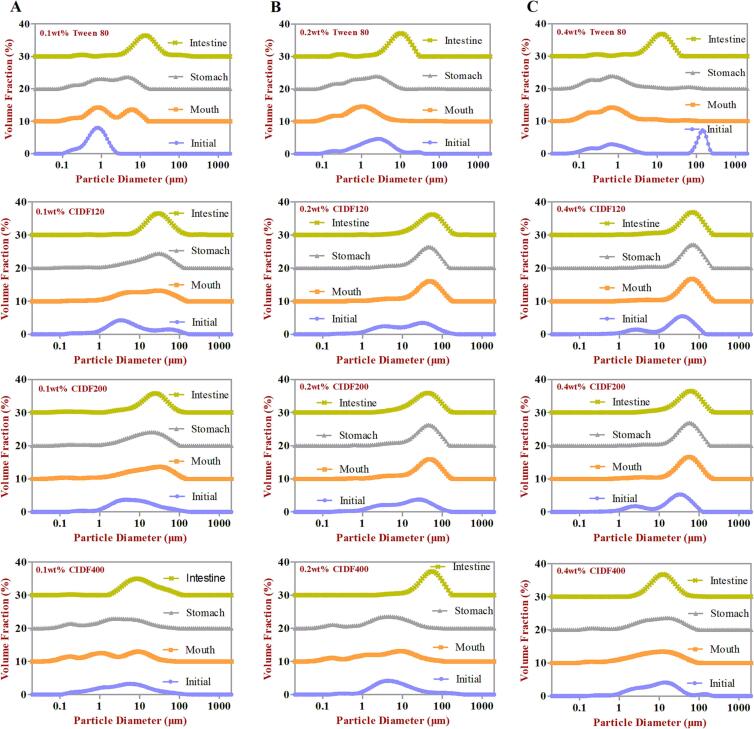
Particle size distribution curves (PSD) of emulsions stabilized by different particle-sized CIDF and Tween80 (control) with various emulsifier concentrations [(a) 0.1 wt%, (b) 0.2 wt%, (c) 0.4 wt%] after being exposed to different phases of a simulated GIT model.

**Fig. 3:**
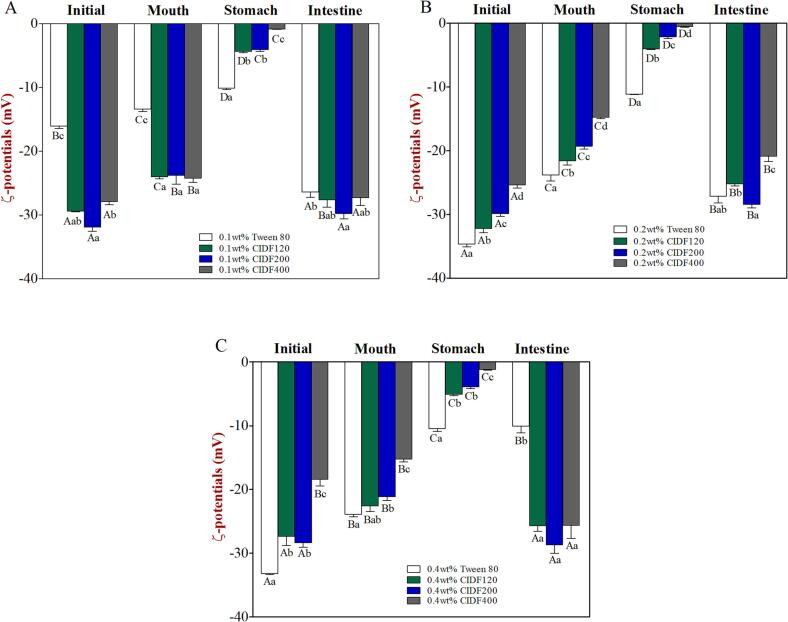
Electrical characteristics (ζ-potentials) of emulsions containing different emulsifiers and various concentration levels [(a) 0.1 wt%, (b) 0.2 wt%, (c) 0.4 wt%] after being exposed to different phases of a simulated GIT model. Different capital letters (*A* to *D*) between different GIT stages (same emulsifier) were significantly different (Duncan, *p* < 0.05), and different lower case letters (*a* to *d*) between different emulsifiers (same GIT stage) were significantly different (Duncan, *p* < 0.05).

**Fig. 4:**
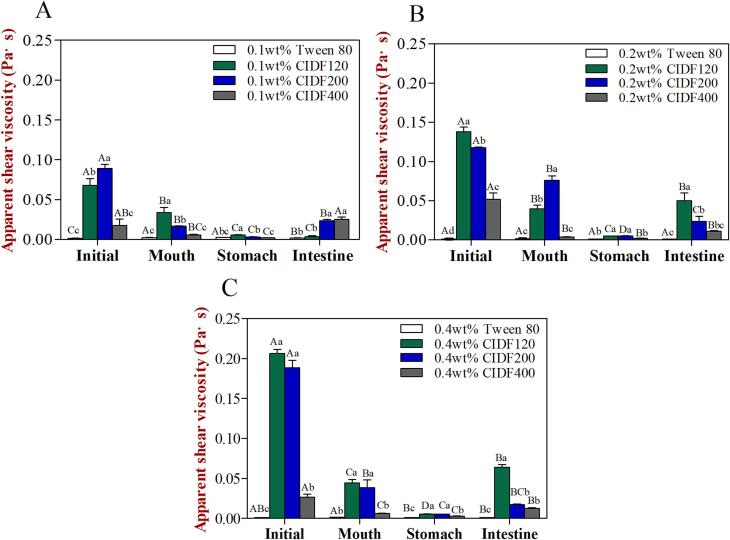
Rheological characteristics (apparent viscosity) of emulsions containing different emulsifiers and various concentration levels [(a) 0.1 wt%, (b) 0.2 wt%, (c) 0.4 wt%] after being exposed to different phases of a simulated GIT model. Different capital letters (*A* to *D*) between different GIT stages (same emulsifier) were significantly different (Duncan, *p* < 0.05), and different lower case letters (*a* to *d*) between different emulsifiers (same GIT stage) were significantly different (Duncan, *p* < 0.05).

**Fig. 5:**
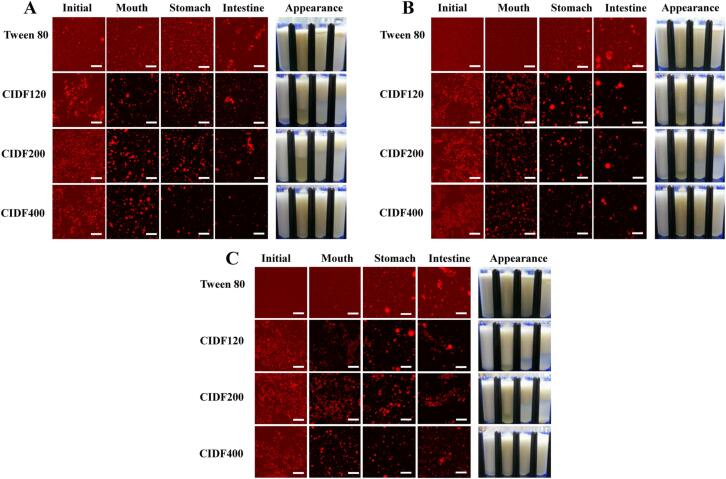
Microstructure and visual appearances of emulsions stabilized by different particle-sized CIDF and Tween 80 (control) and containing various concentration levels [(a) 0.1 wt%, (b) 0.2 wt%, (c) 0.4 wt%] after being exposed to different phases of a simulated GIT model. Scale bar is 50 mm.

In our study, a simulated three-phase GIT model was applied to investigate the lipid digestion behavior of CIDF-stabilized emulsions. Then, mean particle diameter, ζ-potentials, rheological characteristics, and microstructure were characterized after emulsions fabricated under various particle sizes and concentrations of CIDF were passed through the simulated *in vitro* digestion process, as depicted in Fig. 1, Fig. 2, Fig. 3, Fig. 4, Fig. 5.

#### 3.1.1 Initial

For the low emulsifier level (0.1 wt%), the volume-weighted mean particle diameter (*d_43_*) of the initial emulsions stabilized by Tween 80, CIDF120, CIDF200, and CIDF400 were 0.859, 16.495, 15.192, and 10.712 μm, respectively (Fig. 1A), suggesting that mean particle diameter of Tween 80-stabilized emulsion was significantly smaller than that in the CIDF groups. In addition, the Tween 80-stabilized emulsion presented a narrow monomodal particle size distributions (PSD) (Fig. 2A) and the oil droplets seemed to be evenly dispersed, which was confirmed by the confocal microscopy images (Fig. 5A). Conversely, PSDs of CIDF-stabilized emulsions shifted right and the mean particle diameters increased, and a slight aggregation phenomenon occurred in the CIDF-stabilized emulsion droplets (Fig. 5A). The larger particles formed in CIDF-stabilized emulsions may be because Tween 80 had stronger emulsifying property and was more effective at stabilizing emulsions than CIDF (Espinal-Ruiz et al., 2014). Interestingly, as the particle size of CIDF was decreased, mean particle diameters of the emulsion droplets were significantly smaller, which suggested that emulsifying ability of CIDF was greatly enhanced. As the emulsifier level was increased to higher levels (0.2 wt% or 0.4 wt%), the mean particle diameters increased proportionally (Fig. 1) and obvious multimodal appeared in the PSD curves (Fig. 2). This suggested that emulsifier concentration had an obvious impact on the droplets diameter of emulsions, further influencing emulsion stability (Zhang et al., 2020). Moreover, there was no major difference in the microstructures of the initial emulsions except that the oil droplets were more evenly distributed in the continuous phase. This suggests that higher emulsifier concentration showed stronger emulsifying ability and thus prevented emulsions from aggregation, which was further evidenced by the visual appearance of the initial emulsions, especially for the emulsion stabilized by CIDF120 (Fig. 5).

Measurements of the initial ζ-potential values ​​of CIDF-stabilized emulsions were −29.4 mV (CIDF120), −31.9 mV (CIDF200), and −27.9 mV (CIDF400), respectively, indicating a strong negative charged characteristic (Fig. 3A). However, the initial ζ-potential value of Tween 80-stabilized emulsion was −16.1 mV, which was significantly lower than that of CIDF groups. Previous researches proposed that the surface charge of the emulsifier adsorbed on W/O interface was related to the surface charge of the emulsions, and the negative charge of the emulsion stabilized by Tween 80 (nonionic surfactant) was mainly due to the few charged groups adsorbed on the interface. For instance, the existence of OH– group in the water phase and other charged impurities in the emulsifier. (Gomes et al., 2018, Zhang et al., 2015). While negative charge characteristic of the emulsions stabilized by different particle-sized CIDF might be due to the destruction of the fiber structure caused by ultrafine grinding, thus resulting in more exposure of negative charged groups (Costa et al., 2020). As the emulsifier level reached to higher concentration levels (0.2 wt% or 0.4 wt%), initial ζ-potential value of Tween 80-stabilized emulsion significantly increased and no obvious change appeared in the charge values of CIDF-stabilized emulsion, indicating that the emulsifier level had great influences on Tween 80-stabilized emulsion rather than CIDF-stabilized ones.

The apparent viscosity of emulsion stabilized by Tween 80 was markedly lower than that of CIDF-stabilized emulsion in the initial stage (Fig. 4A), which may be due to the thickening effect of CIDF. Previous researches have reported that insoluble dietary fibre (IDF) exhibited high water binding capacity and thickening effect, which may lead to the increase in the viscosity of emulsions (Genovese et al., 2007, Lundberg et al., 2014). In addition, as the particle size of CIDF was reduced, the apparent viscosity of emulsions was decreased accordingly, which might result from the differences in structural properties of CIDF. Our previous study have indicated that the suspension viscosity was significantly decreased with the reducing particle size of CIDF, which may be an important reason for the difference in emulsion viscosity (Feng et al., 2022). Hence, the thickening effect of CIDF might be responsible for the larger particles of CIDF-stabilized emulsions than that of Tween 80-stabilized one. For the higher emulsifier levels (0.2 wt% or 0.4 wt%), the apparent viscosity of all the CIDF-stabilized emulsions was increased significantly, but there were no apparent differences in the apparent viscosity of Tween 80-stabilized emulsion, further confirming the thickening effect of CIDF.

#### 3.1.2 Mouth

At a low emulsifier level (0.1 wt%), the PSDs of emulsions stabilized by CIDF120 and CIDF200 shifted upwards after being subjected to the simulated oral phase, while CIDF400-stabilized emulsions showed the same particle dimensions as the initial emulsions (Fig. 2A). The mean particle diameters of emulsions stabilized by CIDF120 and CIDF200 increased significantly (Fig. 1A), indicated that the oil droplets aggregation might have occurred under the simulated oral phase, as observed from the confocal microscopy images (Fig. 5A). In addition, visual appearances also indicated that those two emulsions were extremely unstable to gravitational separation and divided into two distinct layers (Fig. 5). Those results suggested that the oral conditions promoted the aggregation and flocculation between emulsions as a result of the bridging or depletion flocculation caused by mucin within oral digestive fluids (Sarkar, Goh, & Singh, 2009). Conversely, the mean particle diameter of emulsion stabilized by CIDF400 showed a significant decrease, which may be due to the dilution effect of oral digestive fluids. In addition, the mean particle diameter of Tween 80-stabilized emulsion had undergone a slight increase and less aggregation and flocculation appeared, as could be shown in Fig. 5. The reason for this may be due to the less surfaces on the oil droplets exposed to mucin molecules (Vingerhoeds, Blijdenstein, Zoet, & van Aken, 2005). When the emulsifier concentration was increased to higher levels (0.2 wt% or 0.4 wt%), both of CIDF and Tween 80-stabilized emulsions presented monomodal PSD curves and the mean particle diameters had the similar change trend as those of emulsions in low emulsifier level. Moreover, less aggregation phenomenon of emulsions occurred with the increasing emulsifier concentrations and all the emulsions were evenly dispersed in the continuous phase.

Measurements of the zeta-potential of all the emulsions increased obviously after passage through to the simulated oral period, and the negative charge characteristic was weakened (Fig. 3A). The possible reason for this was probably a result of electrostatic screening caused by ions existing in the simulated oral digestive fluids (Bai et al., 2019, Israelachvili, 2011), or the interactions between positively charged mucin molecules in the digestive fluids and the negatively charged surface of emulsion droplets (Singh & Sarkar, 2011). In addition, the apparent viscosity of Tween 80-stabilized emulsion was increased significantly, while the opposite was true for CIDF-stabilized emulsions. The increase in viscosity of Tween 80-stabilized emulsion may be caused by the presence of mucin in oral digestive fluid. Although the emulsion was diluted by the oral digestive fluids, mucin exhibited a stronger thickening effect on Tween 80-stabilized emulsion that initially possessed a relatively low viscosity. On the contrary, the reduction of apparent viscosity of CIDF-stabilized emulsions may be due to the fact that the thickening effect of CIDF was significantly stronger than that of mucin. Therefore, the apparent viscosity of CIDF-stabilized emulsions decreased after being diluted by oral digestive fluids (Qin, Yang, Gao, Yao, & Mcclements, 2017). Furthermore, the apparent viscosity of CIDF-stabilized emulsions varied due to the differences in the molecular characteristics of CIDF, such as particle size, molecular weight, and conformation (Espinal-Ruiz et al., 2014), and the apparent viscosity of CIDF-stabilized emulsions were increased with higher concentrations of CIDF.

#### 3.1.3 Stomach

After being subjected to the simulated gastric environment, the droplets diameter and PSDs of CIDF and Tween 80-stabilized emulsions showed no obvious differences in the emulsifier concentration of 0.1 wt% (Fig. 1A and 2A). However, observations from visual appearances indicated two distinct layers in the emulsions stabilized by CIDF120 and CIDF200 (Fig. 5A), suggesting that those two emulsions were highly unstable to the simulated gastric environment. Conversely, there were almost no delamination occurring in the CIDF400-stabilized emulsion, which indicated that the emulsion was relatively stable in the simulated gastric environment in the presence of the electrostatic repulsive effect between emulsion droplets (Ozturk, Argin, Ozilgen, & McClements, 2015). In addition, the Tween 80-stabilized emulsion was also relatively stable in the simulated stomach conditions, which may be the result of strong steric repulsion effect (Ozturk et al., 2015).

The negative charge characteristics on the surface of all emulsions were significantly weakened through the simulated gastric phase, especially for CIDF400-stabilized emulsion. The ζ-potential values were almost zero for the CIDF-stabilized emulsions (Fig. 3A), which was in agreement with previously reported results (Bai et al., 2019). The reason for this phenomenon may lie in that a low pH and high ionic strength in the simulated gastric fluid formed electrostatic shielding effects, thus leading to the decreasing zeta-potential value of lipid droplets (Zhang et al., 2015). In addition, no obvious change appeared in the zeta-potential values of the emulsion droplets with higher emulsifier levels (0.2 wt% or 0.4 wt%). Moreover, the apparent viscosity values of CIDF-stabilized emulsions were relatively low after being subjected to simulated gastric environment. The reason for this was that the emulsion samples were further diluted by the simulated gastric fluids, thus leading to significantly decreasing viscosity values in the CIDF-stabilized emulsions. However, the apparent viscosity of Tween 80-stabilized emulsion that initially possessed a relatively low viscosity did not change after being diluted by the simulated gastric fluids. For the high emulsifier levels (0.2 wt% or 0.4 wt%), the apparent viscosity of all the CIDF-stabilized emulsions did not almost change after passage through the simulated gastric phase.

#### 3.1.4 Small intestine

Compared to the simulated stomach stage, the mean particle diameters of all the emulsions increased, especially for the emulsions stabilized by low CIDF levels (Fig. 1), which indicated that the aggregation occurred in the SIF. That was consistent with the results of PSDs (Fig. 2) and confocal microscopic images (Fig. 5). In addition, the mean particle diameters of CIDF-stabilized emulsions were increased with higher levels of CIDF, which was due to a possibility that there was limited surface area of emulsions in the small intestinal conditions resulting in stronger interactions between digesting substances, thus altering the emulsion droplet morphology (Bai et al., 2019). On the one hand, the negative charge on the surface of lipid droplets increased significantly, which might be attributed to the presence of diverse anionic species, such as free fatty acids released, bile acids, and CIDFs. On the other hand, various anions contained in the simulated intestine digestive fluid may play a crucial role in the change of zeta-potential, thereby increasing the negative surface charge characteristic of emulsion droplets. Moreover, some large irregular aggregates in Tween 80-stabilized emulsion were observed from the confocal micrographs (Fig. 5), while there were some undigested oil droplets occurring in the CIDF-stabilized emulsions, suggesting that CIDF might have retard the lipid digestion to some extent.

The apparent viscosity of the emulsions stabilized by CIDF200 and CIDF400 increased significantly and the viscosity of other groups showed a slight decrease. The reason why the viscosity of CIDF-stabilized emulsions with smaller particle size of CIDF were increased significantly might be due to the change of its physicochemical properties treated by ultrafine grinding (Feng et al., 2022). When the emulsifier concentration reached to 0.2 wt% or 0.4 wt%, the apparent viscosity values of Tween 80-stabilized emulsion had no apparent change, indicating its concentration independence. However, the viscosity of emulsions stabilized by different particle-sized CIDF increased significantly with the increasing concentration, suggesting that the thickening effect of CIDF was strengthened, which might bring about stronger impact on the lipid digestion behavior of the emulsions.

### 3.2 Influence of particle sizes and concentrations of CIDF on lipid digestion of emulsions

Generally, the lipolysis reaction occurring in the small intestinal phase can generate various substances, including free fatty acids (FFAs), monoacylglycerols, and hydrogen ions, thus leading to a gradual drop of pH value in the system. Therefore, to maintain a constant neutral pH of the system. NaOH solutions (0.1 M) were added dropwise by an automatic titration device, and the lipolysis extent was assessed by recording the volume of NaOH consumed over time (Deloid et al., 2018).

**Fig. 6:**
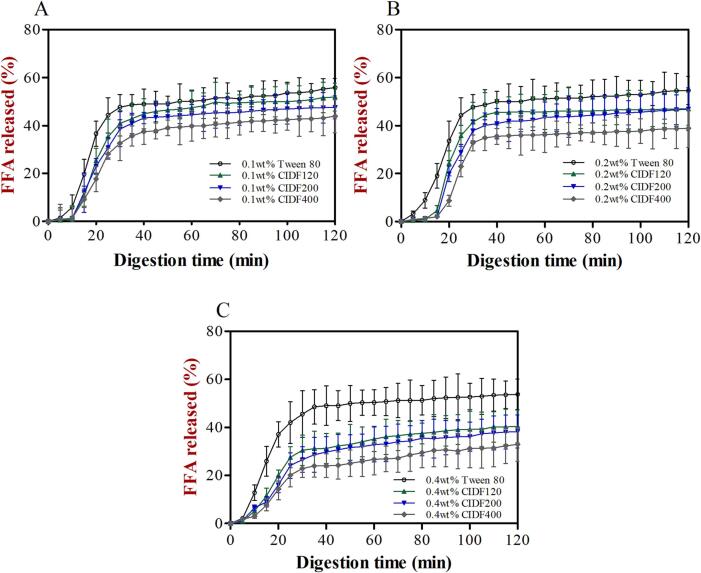
Percent of total available free fatty acids (FFAs) released from emulsions stabilized by different particle-sized CIDF and Tween 80 (control) and containing various concentration levels [(a) 0.1 wt%, (b) 0.2 wt%, (c) 0.4 wt%] after being exposed to different phases of a simulated GIT model.

The lipid hydrolysis profiles of Tween 80 and CIDF-stabilized emulsions were depicted in Fig. 6, and the initial and total extent of FFAs released were presented in Fig. S2 (Supplementary data). For the low concentration group (0.1 wt%), the lipid digestion rate curves of all emulsions were very similar, with a rapid generation of large amounts of FFA within the first 30 min, followed by a gradual flattening of the release rate, indicating that lipid digestion reaction mainly occurred in the initial stage. In addition, in the first 30 mins of digestion, the release rate of FAA was relatively slow at the beginning, and then increased sharply, which may be due to the presence of CIDF on the surface of the emulsions to form a physical barrier, further blocking the access of lipase to lipids. However, with the prolonger of digestion time, CIDFs on the surface of the emulsions were gradually replaced from lipid droplets by bile salts in the intestinal fluid to start the lipid hydrolysis reaction (Bai et al., 2019). For Tween 80-stabilized emulsion, it could be seen that lipids were almost completely hydrolyzed, which was in line with the microscopic images (Fig. 5). Moreover, the digestion rate and extent of emulsions stabilized by different particle-sized CIDF were always lower than that of emulsion stabilized by Tween 80, which showed that CIDFs were possible to inhibit the lipid hydrolysis process. When the emulsifier concentrations were increased to higher levels, this inhibitory effect seemed to be more pronounced. Among those CIDFs, the CIDF400 showed the strongest retarded effect on the lipid digestion rate and extent, and the maximum inhibition extent of lipolysis for CIDF400-stabilized emulsion was 38.77% in contrast to Tween 80-stabilized one (Fig. S2B). That may be attributed to the fact the considerable thick protective coatings formed on the surface of the emulsions, thus reducing the possibility of lipase to absorb to lipid droplets (Qin et al., 2016). On the other hand, CIDF might bind to bile salts to interfere with the lipolysis process (Hu, Li, Decker, Xiao, & McClements, 2010). Previous studies have suggested that the role of bile salts was to remove FAAs accumulated on the surface of the lipid droplets. When bile salts interacted with other substances (such as CIDF), the released FAAs gradually aggregated around the lipid droplets to further block the hydrolysis of internal lipids, which in turn reduced the lipolysis rate and extent (Devraj et al., 2013, Qin et al., 2016, Winuprasith et al., 2018). In addition, the concentration of CIDF may have an obvious impact on the lipid digestion rate and extent, and this effect was enhanced with higher emulsifier levels. The reason for this was that the high emulsifier levels tended to correspond to highly viscous aqueous phase of emulsions, which further restricted the diffusion and movement of some key substances (such as bile salts and lipase) in the small intestine phase, finally inhibiting lipid digestion rate and extent (Qin et al., 2016, Zhang et al., 2015). Interestingly, in this study, the factors influencing the lipid digestion mainly included two aspects, namely particle size of CIDF and viscosity of emulsion system, and our results indicated that the particle size seemed to have a greater impact than viscosity property (Fig. 6). Therefore, further investigation was needed to explain why the influence was dominated by particle size of CIDF in our study.

## 4 Conclusions

In this study, the influences of different particle-sized CIDF on lipid digestion behavior were investigated by measuring the changes of physicochemical characteristics, such as mean droplet diameter, zeta-potential, rheological property and microstructure of CIDF-stabilized emulsions during the simulated GIT model. Our results suggested that different particle-sized CIDF could delay the lipid digestion rate and extent due to the droplets aggregation and flocculation caused by the change of zeta-potential and apparent viscosity of emulsions. In addition, the CIDF400 had the strongest inhibition effect on the lipid digestion extent, and the maximum inhibition extent of lipolysis was 38.77% for CIDF400-stabilized emulsion at the emulsifier concentration of 0.4 wt%, compared with Tween 80-stabilized one. Moreover, the lipolysis rate and extent were more obviously reduced when presented at higher CIDF levels. Finally, we preliminarily concluded that CIDF might affect the rate and extent of lipid digestion through two possible approaches: (1) protective layers formed around the droplets blocked the contact of lipase with internal lipids; (2) the increasing viscosity of CIDF- stabilized emulsions limited the transportation of some substances in the simulated small intestine digestion. This study may provide new sustainable perspectives for the application of CIDF and the development of functional foods that reduce weight and promote health and wellness.

## Funding

This study was financially supported by HZAU-AGIS Cooperation Fund (SZYJY2022013) and the Fundamental Research Funds for the Central Universities (2662022JC003).

## CRediT authorship contribution statement

**Ben Yu:** Conceptualization, Investigation, Methodology, Data curation, Formal analysis, Writing – original draft. **Qianqian Chen:** Data curation, Formal analysis. **Joe M. Regenstein:** Conceptualization, Supervision, Writing – review & editing. **Changwen Ye:** Funding acquisition, Writing – review & editing. **Lufeng Wang:** Supervision, Validation, Funding acquisition, Writing – review & editing.

## Declaration of Competing Interest

The authors declare that they have no known competing financial interests or personal relationships that could have appeared to influence the work reported in this paper.

## Data Availability

Data will be made available on request.
