# Supplementary material for: The lipid digestion behavior of oil-in-water emulsions stabilized by different particle-sized insoluble dietary fiber from citrus peel
Source: Food Chem X. 2023 Aug 9;19:100831. doi: 10.1016/j.fochx.2023.100831 (PMC10534149; doi:10.1016/j.fochx.2023.100831)
Supplement: Supplementary data 1 [file mmc1.docx]

**Fig. S1.** Apparent viscosity of different particle-sized insoluble dietary fiber from citrus peel (CIDF, 3.0 wt%).


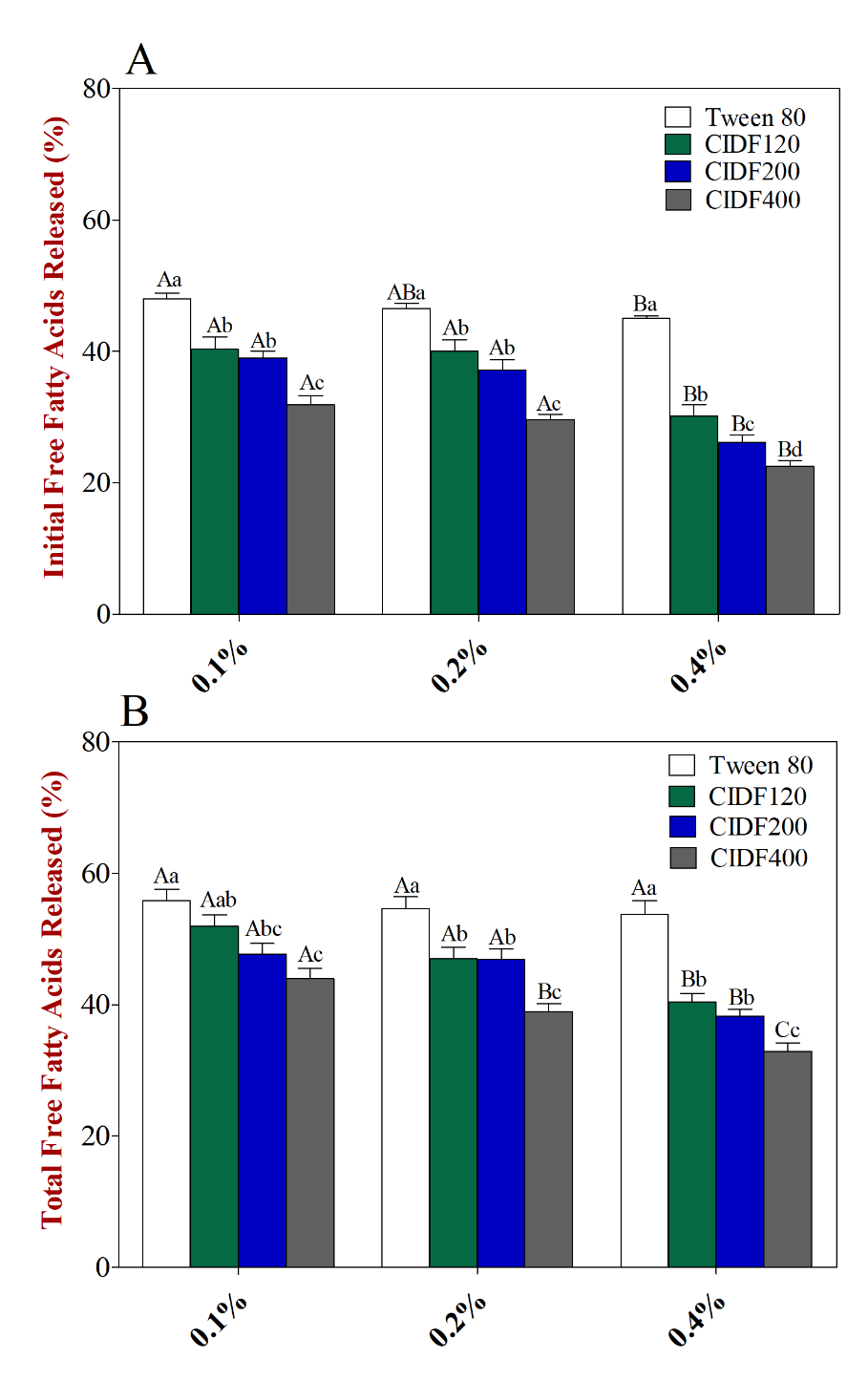


**Fig. S2.** (a) Initial and (b) total percentage of free fatty acids (FFAs) released from emulsions stabilized by different particle-sized CIDF and Tween 80 (control) and containing various concentration levels [(a) 0.1 wt%, (b) 0.2 wt%, (c) 0.4 wt%] after being exposed to different phases of a simulated GIT model. Different capital letters (*A* to *C*) between different emulsifier concentrates (same emulsifier) were significantly different (Duncan, *p* < 0.05), and different lower case letters (*a* to *d*) between different emulsifiers (same emulsifier concentrate) were significantly different (Duncan, *p* < 0.05).
